# Supplementary material for: Embryonic and foetal expression patterns of the ciliopathy gene CEP164
Source: PLoS One. 2020 Jan 28;15(1):e0221914. doi: 10.1371/journal.pone.0221914 (PMC6986751; doi:10.1371/journal.pone.0221914)
Supplement: S1 Table — (DOCX) [file pone.0221914.s001.docx]

| **Antigen** | **Host Species** | **Species Reactivity** | **Source** | **Working Dilution** |
| --- | --- | --- | --- | --- |
| CEP164 | Rabbit | Human | Protein Atlas (HPA037606) | 1/700 |
| PAX6 | Rabbit | Human, Mouse | Covance (PRB-278-P-100) | 1/700 |

**S1 Table. Working dilutions of primary antibodies used for immunohistochemistry of human tissues**
