# Supplementary material for: Embryonic and foetal expression patterns of the ciliopathy gene CEP164
Source: PLoS One. 2020 Jan 28;15(1):e0221914. doi: 10.1371/journal.pone.0221914 (PMC6986751; doi:10.1371/journal.pone.0221914)
Supplement: S2 Table — (DOCX) [file pone.0221914.s002.docx]

| **Human Development**  **Stage** | **Murine Development**  **Stage** | **Stage of Renal Development** |
| --- | --- | --- |
| Early Development 3.5-7 PCW | Early Development E7.5-E13.5 | - Rostro-caudal migration of the pronephric duct. - Pronephros interacts with the adjacent mesoderm to form the mesonephric duct. - Non-functional pronephri degenerates quickly. - Mesonephric duct extends rostro-caudally, joining the cloaca. - Ureteric bud is formed, which interacts with adjacent metanephric mesenchyme, causing further branching. - Mesonephros starts to degenerate. - Metanephric mesenchyme condense, undergo mesenchymal to epithelial transition forming renal vesicles with defined apical-basal polarity. - Invagination of renal vesicles causes formation of the comma-shaped body. - Further invagination leads to development of the S-shaped renal bodies. |
| 7-8 PCW | E13.5 – E16.5 | - The S-shaped body forms a glomeruli cleft, containing podocyte progenitors. - Distal end of S-shaped body fuses with the mesonephric branching ureteric bud. - Blood vessels progenitors invade, initiating the formation of vascular structures. |
| 8-9 PCW | E16.5 - E17.5 | - Glomeruli capsules are developing. - Clear structures of the nephron can be seen including Bowmans capsules, glomerular tufts, collecting ducts, and distinguishing proximal and distal tubules. |
| 14-15 PCW | E18.5 | - Loop of Henle ascending and descending segments are present. - Ureter is present. |
| 35-36 PCW | P2-P4 | - Nephrogenesis ceases. |
| 6 months of age | P21 | - Kidney is mature |

**S2 Table. Comparison of human and murine kidney developmental timeline**
