# Supplementary material for: Embryonic and foetal expression patterns of the ciliopathy gene CEP164
Source: PLoS One. 2020 Jan 28;15(1):e0221914. doi: 10.1371/journal.pone.0221914 (PMC6986751; doi:10.1371/journal.pone.0221914)
Supplement: S3 Table — (DOCX) [file pone.0221914.s003.docx]

| **Human Development Stage** | **Murine Development Stage** | **Stage of Retinal Development** |
| --- | --- | --- |
| 6-7 PCW | E12.5 | - Retinal pigment epithelium (RPE) has formed as a pseudostratified columnar epithelial cell layer. - Neuroblastic epithelium is also present, which is separated by a transient fibre Layer of Chievitz (human). - Eyelids are developing. |
| 7-8 PCW | E15.5 | - The Layer of Chievitz is lost. - Nerve fibre cell layer (NFL) is formed. - Neuroblastic layer thickens. |
| 8-9 PCW | E18.5-P0.5 | - Inner plexiform layer (IPL) develops, separating the neuroblastic layer into the developing ganglion cell layer (GCL) (inner section) and outer neuroblastic cell layer (ONBL) (outer section). - Nerve fibre cell layer (NFL) forms a discrete layer. - Photoreceptor cone precursors in the outer neuroblastic cell layer (ONBL) start to differentiate. |
| 10-12 PCW | P5.5 | - Outer neuroblastic layer differentiates into cone precursors and inner nuclear cells. |
| 12-13 PCW |  | - First blood vessels appear in the nerve fibre cell layer. |
| 13-15 PCW | P15 | - Rods are now present in the photoreceptor cell layer. - Inner plexiform layer, and ganglion cell layer are still developing. - Outer plexiform layer is developing, forming inner and outer nuclear cell layers. - Cilia project from photoreceptor cell layer to the retinal pigment epithelium layer. - Muller cells are present in the ganglion cell layer and the developing inner nuclear cell layer. - Bipolar cells are present in the developing inner nuclear cell layer. - Eyes open (mice). |
| 18 PCW | P18 | - Rods and cones are now distinguishable. - Retinal pigment epithelium is now a single layer of cuboidal cells. - All retinal layers are present. |
| 26 PCW |  | - Eyelids open (human) |
| 12 months old (approx.) | P21 (approx.) | - Retina is fully functional |

**S3 Table. Comparison of human and murine retina developmental timeline**
