# Supplementary material for: Embryonic and foetal expression patterns of the ciliopathy gene CEP164
Source: PLoS One. 2020 Jan 28;15(1):e0221914. doi: 10.1371/journal.pone.0221914 (PMC6986751; doi:10.1371/journal.pone.0221914)
Supplement: S4 Table — (DOCX) [file pone.0221914.s004.docx]

| **Human Development Stage** | **Murine Development Stage** | **Cerebellar Morphology** | **Cerebellar Cell Development** | **Cerebellum Foliation Development** |
| --- | --- | --- | --- | --- |
| 4-6 PCW | E9.5 | - The tuberculum cerebelli thickens caudally and laterally (band of tissue at the dorsolateral section of the alar plate). - Cerebellar bulge forms (subsequently develops into the corpus cerebelli). - Rhombic lip forms. |  |  |
| 6 PCW |  |  | - Neurone progenitors migrate from the ventricular zone. |  |
| 7-8 PCW |  | - Cerebellar plate thickens. - Preliminary cerebellar hemispheres are formed from expansion of rhombic lip with the alar plate. | - Dentate nucleus progenitors migrate from the ventricular zone and rhombic lip. - Developing external granular cell layer extends from rhombic lip to the dorsum of the cerebellar bulge. |  |
|  | E13.5 |  | - Granular cell layer interneurons differentiate. |  |
| 10 PCW | E14.5 | - Cerebellar hemispheres join at the midline. | - Molecular layer interneurons differentiate. |  |
| 11 PCW |  | - Cerebellum continues to grow, with the thickness equivalent to the midbrain. - Cerebellar vermis is formed. |  |  |
| 11-12 PCW | E17.5 |  |  | - Primary cerebellar fissures develop. (In the mouse this is 3 lobes) |
| 12-13 PCW |  | - Cerebellum growth and cell migration, causes the cerebellum to be situated over the fourth ventricle. |  |  |
| 13-15 PCW | E18.5 |  | - Purkinje cell layer established. | - 4 principle cerebellar fissures present (preculminate, primary, secondary and posterolateral. - 5 cardinal lobes present (anterobasal, anterodorsal, central, posterior, inferior lobe). - These cardinal lobes start dividing. |
|  | P0.5 |  | - Unipolar brush cells migrate to the inner granular cell layer. |  |
| 15-16 PCW | P0.5-P3.5 | - Dentate nucleus is present. | - External granular cells migrate to form inner granular layer and differentiate. | - Cerebellar fissures deepen. |
| 20-28 PCW |  |  | - 5 layers present; external granular cell layer, molecular cell layer, purkinje cell layer, internal granular cell layer and lamina dissecans (human). |  |
| 32 PCW | P5 |  | - Lamina dissecans is lost (humans). |  |
|  | P7 |  |  | - Cardinal layers have divided, forming lobules. |
|  | P14-P16 |  | - Cell layers continue to differentiate and develop. | - Structure of the adult folia can be seen by now (mouse). |

**S4 Table. Comparison of human and murine cerebellar development timeline.**
