# Supplementary figures and images for: Embryonic and foetal expression patterns of the ciliopathy gene CEP164
Source: PLoS One. 2020 Jan 28;15(1):e0221914. doi: 10.1371/journal.pone.0221914 (PMC6986751; doi:10.1371/journal.pone.0221914)

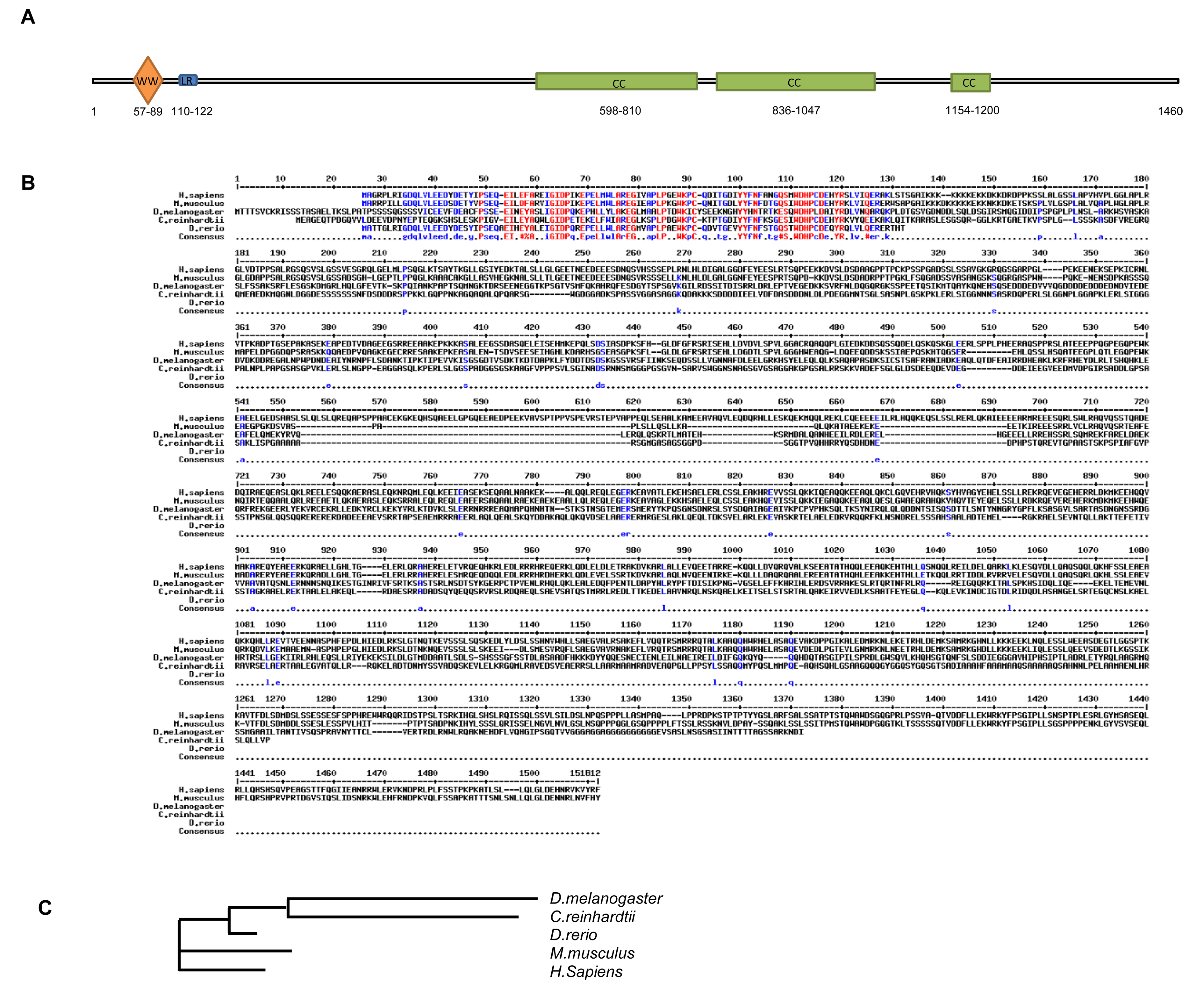

Supplement: S1 Fig — Predicted human CEP164 protein domains of the common 1460bp isoform; tryptophan-tryptophan (WW) domain conserved with two Tryptophan (W) residues, Lysine-rich repeat (LR) and predicted coiled-coil (CC) domains. Values marked are amino acid number (A). Sequence alignment of human CEP164 and its orthologs in M.musculus, D.melanogaster, C. reinhardtii and D rerio (B). Unrooted phylogenetic tree of CEP164 orthologs (C). (TIF) [file pone.0221914.s005.tif]

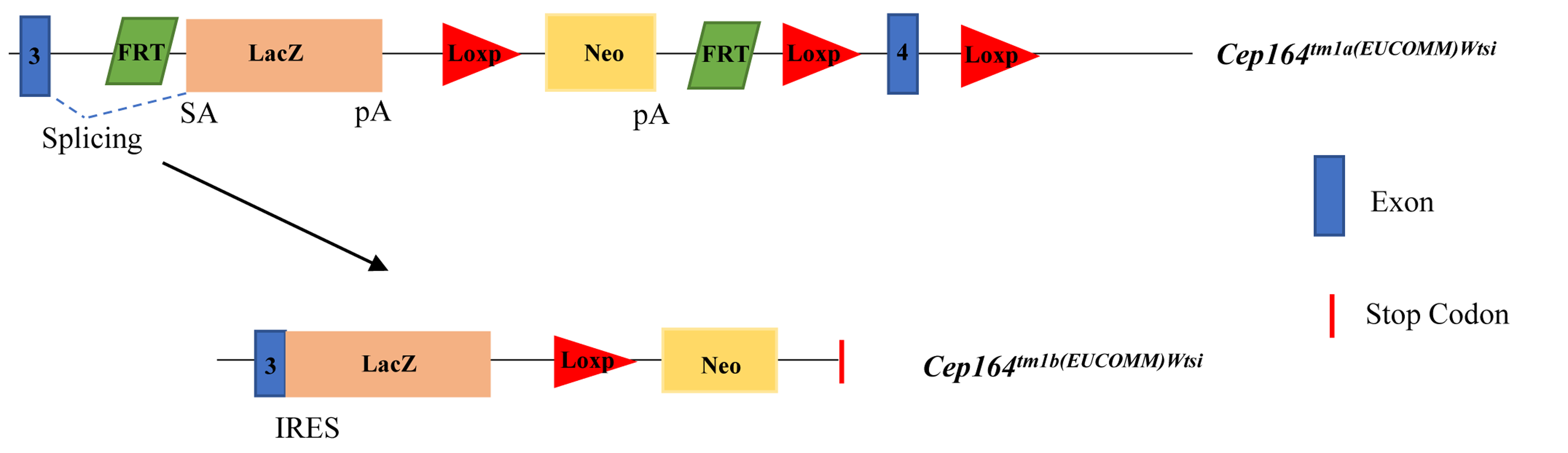

Supplement: S2 Fig — Upon pre-mRNA splicing of Cep164tm1a(EUCOMM)Wtsi, exon 3 splices into the splice acceptor (SA) of the LacZ cassette, causing a frameshift and subsequent formation of a premature termination codon, this forms the tm1b allele. The LacZ has an internal ribosomal entry site (IRES), and thus the LacZ fusion gene acts as a reporter gene for Cep164. Figure adapted from MRC Harwell International Mouse Phenotyping Consortium. Internal ribosomal entry site (IRES), splice acceptor (SA), polyadenylation site (pA). (TIF) [file pone.0221914.s006.tif]

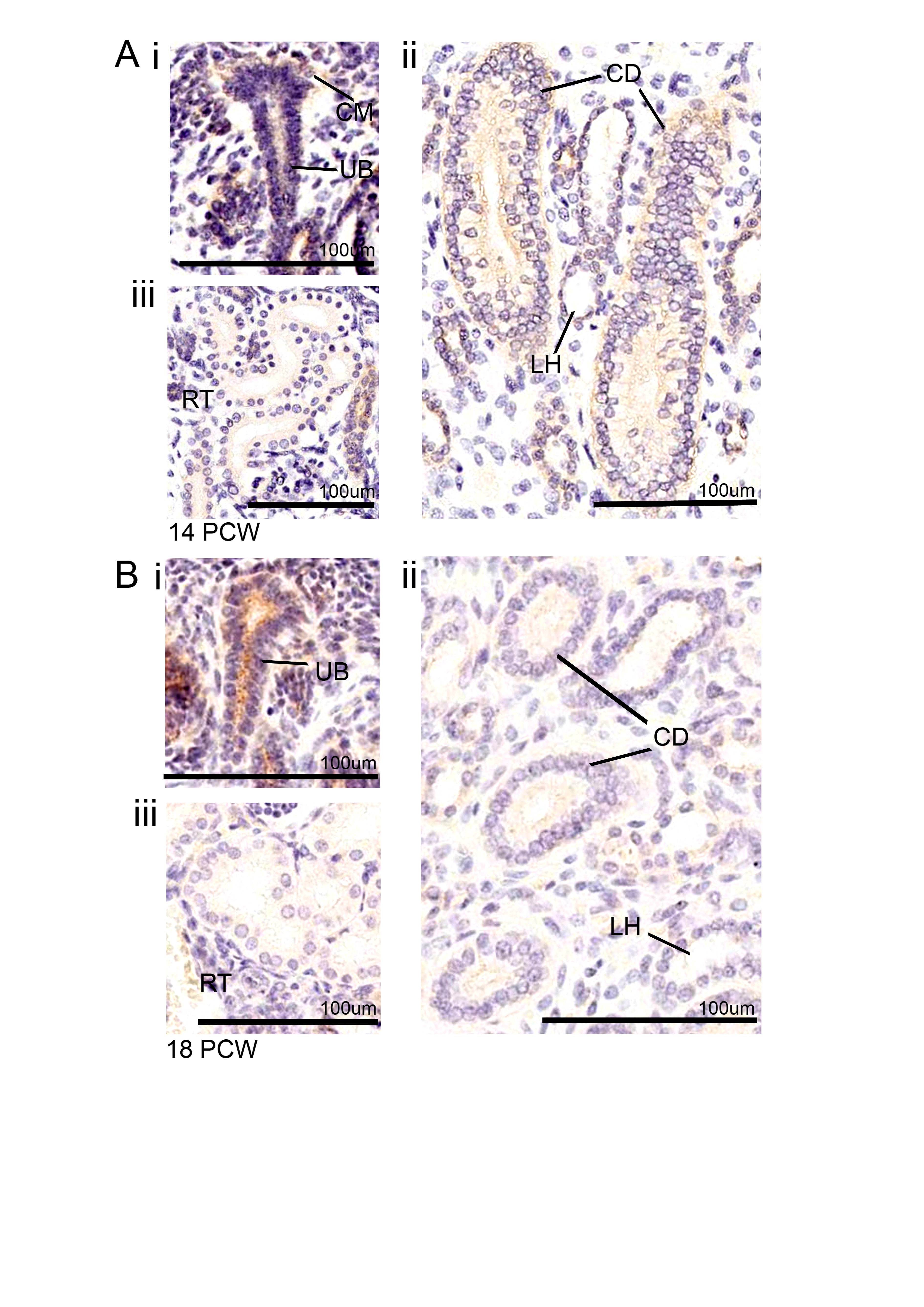

Supplement: S3 Fig — Human kidney 14 PCW (A), 18 PCW (B). CEP164 is expressed strongly at the apical membrane of the ureteric bud (A.I, B.I) which is maintained in the collecting duct, with CEP164 expression also at the basolateral membrane (A.II,B.II). CEP164 is also expressed strongly at the renal tubule apical membrane (A.III, B.III). Cap mesenchyme (CM), collecting duct (CD), loop of Henle (LH), renal tubule (RT), ureteric bud (UB). (TIF) [file pone.0221914.s007.tif]

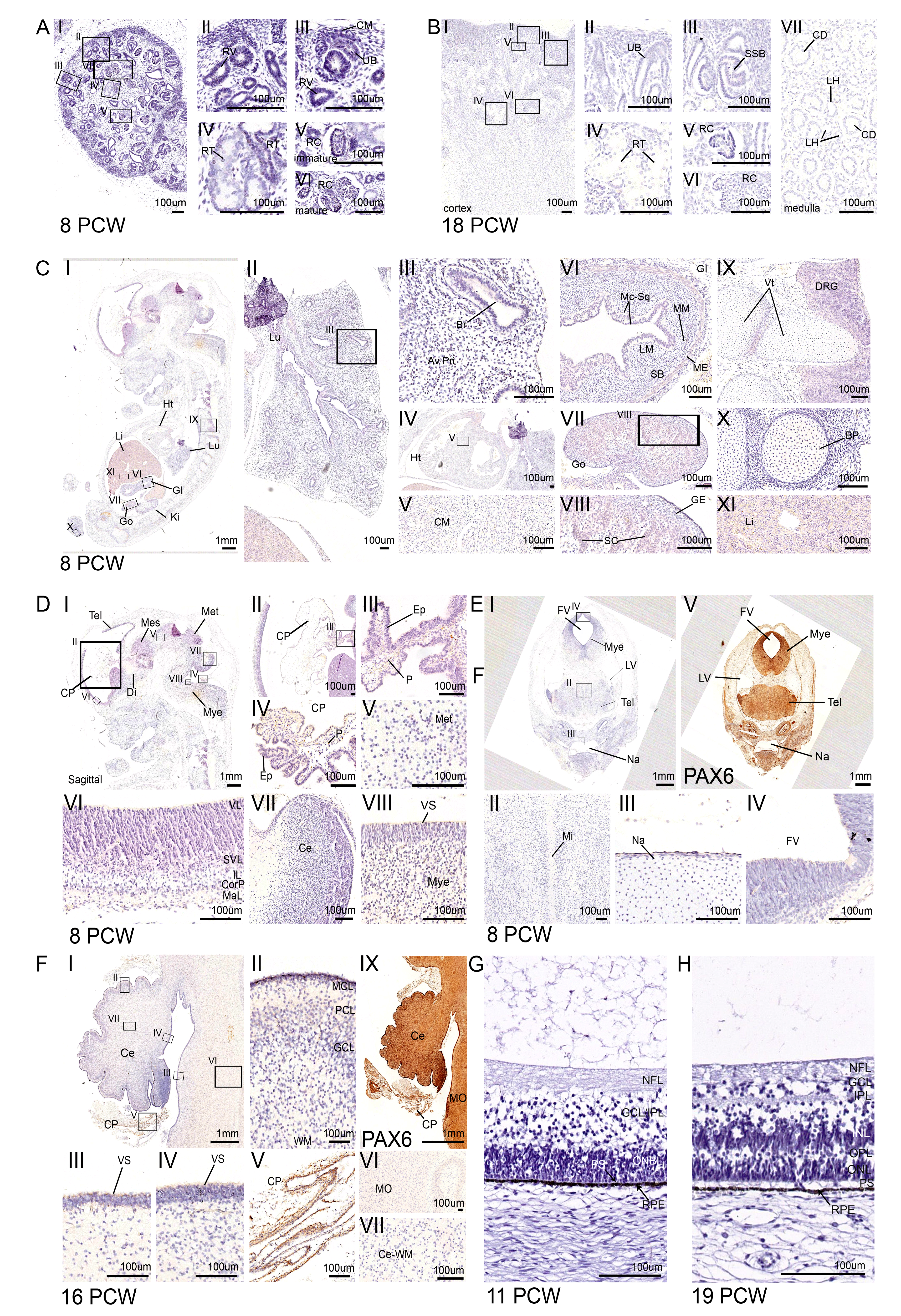

Supplement: S4 Fig — No primary antibody controls in the human 8 PCW kidney (A) and human 18 PCW kidney (B). Renal vesicles, comma-shaped vesicles, s-shaped body, ureteric bud and cap mesenchyme demonstrate no endogenous peroxidase staining (A.I.II.III, B.I.II.III). Renal tubule and the renal corpuscle (A.IV.V.VII, B.IV.V.VI), loop of Henle and collecting ducts (B.VII) also demonstrate no endogenous peroxidase staining. No primary antibody controls 8 PCW embryo (C). Lung, including the bronchioles and alveoli (C.I.II.III) and cardiomyocytes (C.I.IV,V) show no endogenous peroxidase staining. There is very weak endogenous staining in the Muscularis Externa (ME) of the gastrointestinal tract (C.VI) and the seminiferous cord of the gonads (C.VII.VIII). Dorsal root ganglia (C.IX) and bone primordia (C.VX) demonstrate no endogenous staining. However, the hepatocytes of the liver have endogenous peroxidase staining (C.IX), which means it cannot be determined if CEP164 is expressed in hepatocytes. The developing brain no primary controls, 8PCW (D, E) and 16 PCW (F). The developing brain (D.I, E.I) including the telencephalon (D.VI), metencephalon (D.V), midline (E.II) and myencephalon (D.VIII) do not demonstrate endogenous peroxidase staining. The neuroepithelium surrounding the brain ventricles (D.VIII, E.III.IV, F.I.III.IV) alongside the choroid plexus ependymal cells (D.II.III.IV, F.I.V) and choroid plexus pia matter (D.III.IV, F.I.V) do show some weak endogenous peroxidase staining. The molecular cell layer, purkinje and ganglion cell layers of cerebellum (F.I.II) and cerebellar white matter (F.VII) do not show endogenous staining. The ventricular surface (F.III.IV), medulla oblongata (F.VI) and choroid plexus at 16 PCW (F.V) show weak CEP164 staining. PAX6 positive control antibody (E.V, F.IX). The developing retina no primary controls, 11 PCW (G), and 19 PCW (H), both demonstrate no endogenous staining. Alveoli primordia (Av Pri), bone primordia (BP), bronchiole (Br), cap mesenchyme (CM), [file pone.0221914.s008.tif]

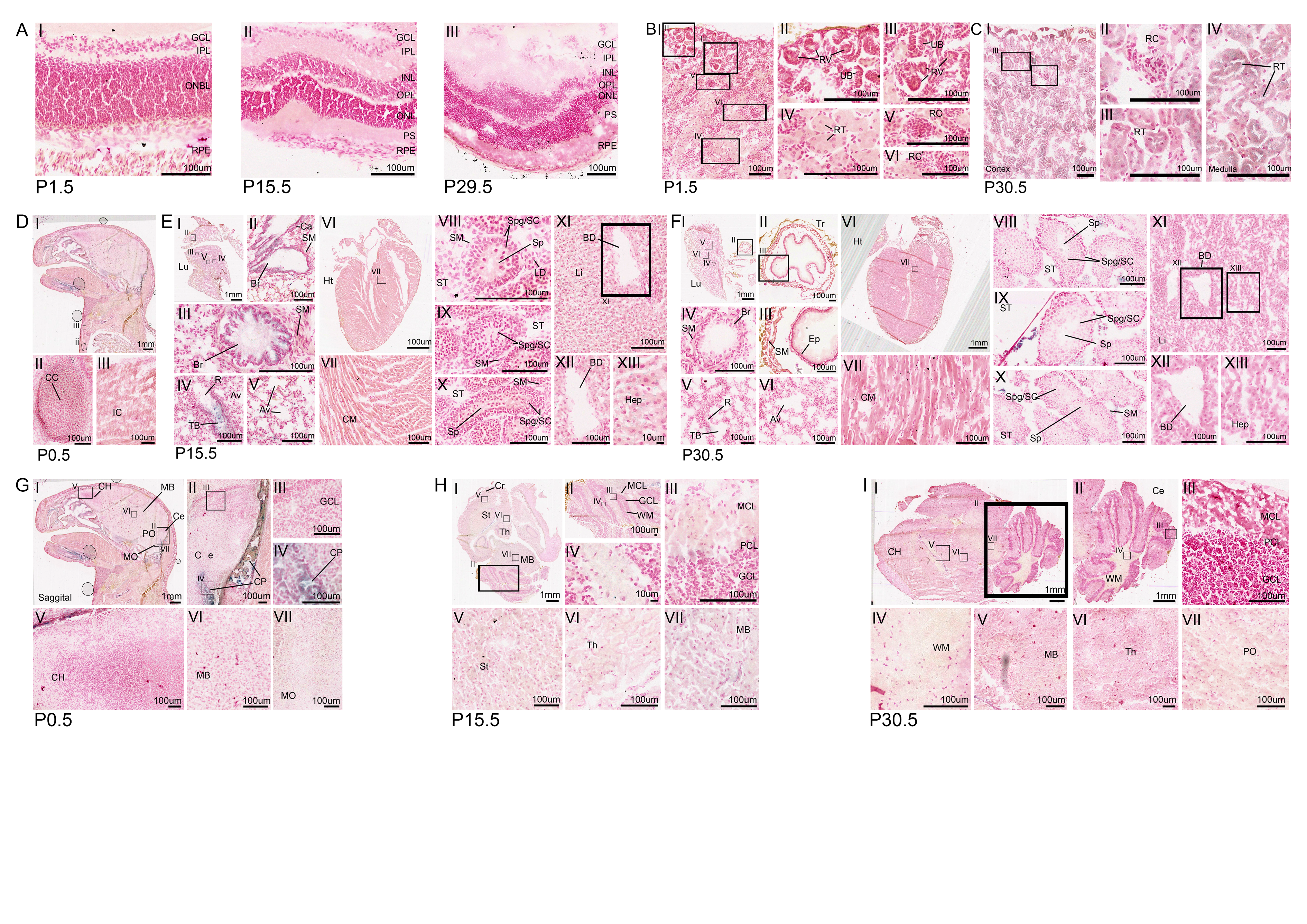

Supplement: S5 Fig — Murine retina wildtype controls (A). Murine kidney wildtype controls (B-C), murine secondary tissues wildtype controls (D-F), and murine cerebellar tissues (G-I). Retina at P1.5 (A.I), P15.5 (A.II), and P29.5 (A.III) demonstrate no endogenous beta galactosidase staining. Renal vesicles (B.I.II.III), ureteric bud (B.III), renal tubules (B.IV) and renal corpuscle (B.V.VI) at P1.5 do not show any endogenous staining. Renal tissue at P30.5 have low endogenous beta galactosidase expression in the renal tubules (C.I.III.IV), but not in the renal corpuscle (C.II). Developing brain (D.I), costal cartilage (D.II) and intercostal muscle (D.III) at P0.5 do not show endogenous beta galactosidase staining. The P0.5 lung bronchioles (E.I.II.III.IV) demonstrate weak endogenous beta galactosidase staining, but not the alveoli (E.V). The P30.5 murine lung trachea, bronchioles and alveoli (F.I.II.III.IV.V.VI) do not show endogenous beta galactosidase staining. Murine cardiomyocytes (E.VI.VII, F.VI.VII), developing testes including the spermatogonia, spermatocytes, spermatids and smooth muscle cells (E.VIII.IX.X, F.VIII.IX.X) do not have endogenous staining. There is also no endogenous staining in the hepatocytes of the liver, hepatic portal vein (E.XI.XII.XIII, F.XI.XII.XIII). Murine brain cerebral hemisphere (G.I.V, H.I.V, I.I.), the midbrain (G.VI,H.VII,I.V), the striatum (H.V,) the thalamus (H.VI,I.VI) the pons and the medulla oblongata (G.I.VII, H.I.VII, I.I.VII) all demonstrate no endogenous staining. The choroid plexus shows some weak endogenous beta galactosidase expression (G.I.II.IV) at P0.5. In the developing the cerebellum, the ganglion cell layer, molecular cell layer, purkinje cell layer and white matter of the cerebellum (G.II.III, H.II.III.IV, I.II.III.IV) demonstrate no endogenous beta galactosidase staining. Alveoli (Av), bronchiole (Br), cardiomyocyte (CM), cartilage (Ca), cerebellum (Ce), cerebral hemisphere (Ch), cerebrum (Cr), choroid plexus (CP), connective tiss [file pone.0221914.s009.tif]

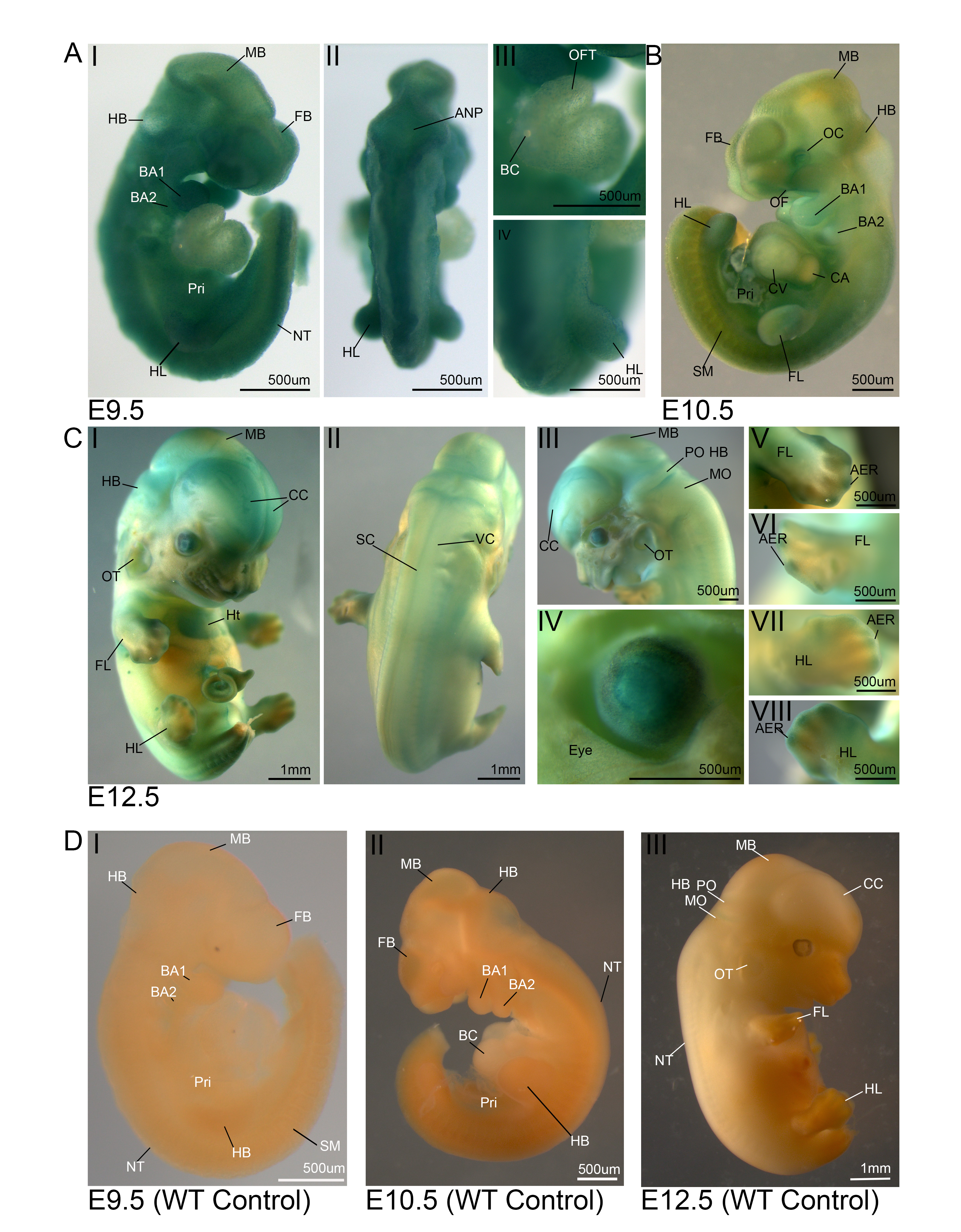

Supplement: S6 Fig — At E9.5 (A), Cep164 widespread expression is seen, including the branchial arches, developing forebrain, midbrain and hindbrain (A.I). There is also Cep164 expression seen in the developing neural tube, including the neuroepithelium surrounding the anterior neuropore (A.II). There is Cep164 expression seen in the developing heart including the central ventricle, bulbous cordis and outflow tract (A.III), as well as the developing limb buds (A.IV). This Cep164 expression pattern is maintained at E10.5, with Cep164 expression also seen in the optic cup and olfactory pit (B). At E12.5 Cep164 expression is widespread but is defined in the spinal cord, vertebrae, cerebral cortex, midbrain, hindbrain, pons and medulla oblongata, otocyst and heart (C.I.II.III). Cep164 expression is seen in the retina (C.IV) and at the tips of the developing digits, where the apical ectodermal ridge is present (C.V.VI.VII.VIII). WT littermates do not show endogenous beta galactosidase expression at E9.5 (D.I), E10.5 (D.II), and E12.5 (D.III). Anterior nucleopore (ANP), apical ectodermal ridge (AER), branchial arch (BA), bulbous cordis (BC), cerebral cortex (CC), common atria (CA), common ventricle (CV), forebrain (FB), forelimb bud (FL), heart (Ht), hindbrain (HB), hindlimb bud (HL), medulla oblongata (MO), midbrain (MB), neural tube (NT), olfactory pit (OF), optic cup (OC), organ primordia (Pri), otocyst (OT), outflow tract (OFT), pons (PO), somites (SM), spinal cord (SC), vertebral column (VC). (TIF) [file pone.0221914.s010.tif]

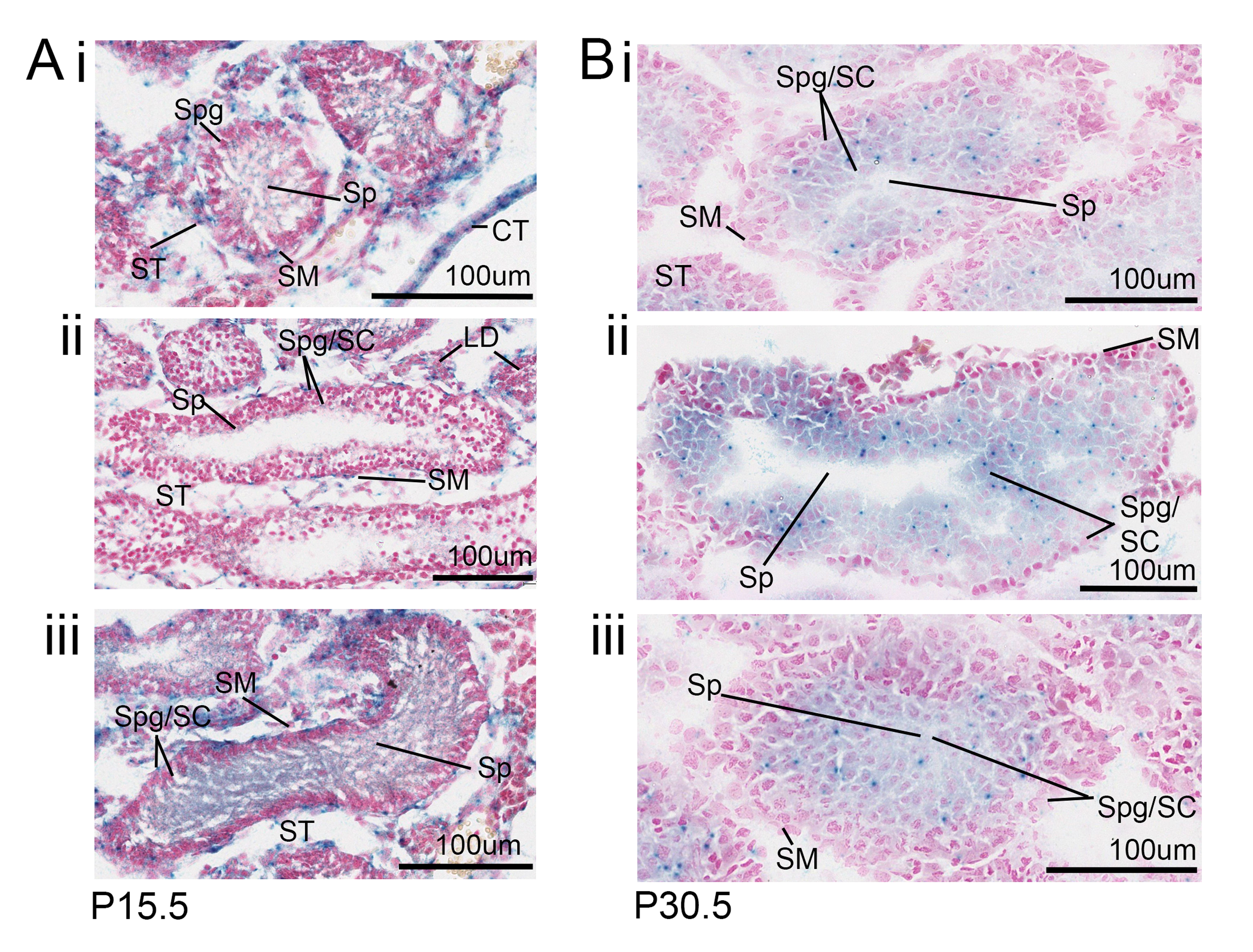

Supplement: S7 Fig — P15.5 murine testes (A) P30.5 murine testes (B). Cep164 is expressed in the connecting tubules and leydig cells (A.I.II). In the seminiferous tubule, Cep164 is expressed in the smooth muscle cells, spermatogonia and spermatocytes (A.B). Cep164 is expressed most strongly in the spermatid, tails (A.III). Connecting tubule (CT), Leydig cells (LD), seminiferous tubule (ST), smooth muscle cells (SM), spermatocytes (SC), spermatogonia (Spg), sperm (Sp). (TIF) [file pone.0221914.s011.tif]

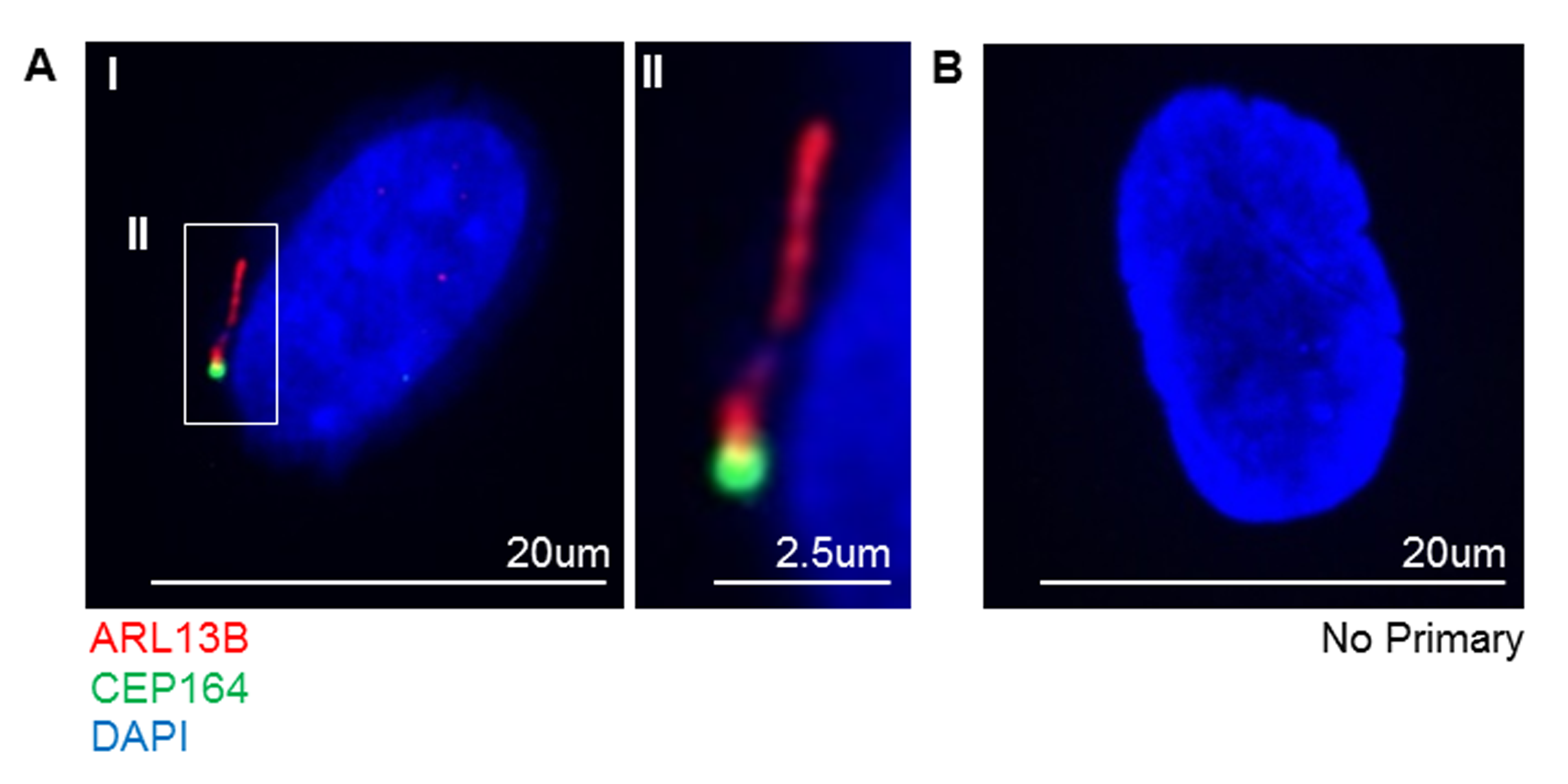

Supplement: S8 Fig — Representative image of human urine derived renal epithelial cells (hURECs) with rabbit anti- CEP164 staining (Human Protein Atlas, HPA37606) (A). CEP164 (green) can be seen at the base of the primary cilium, stained with mouse anti-ARL13B (Proteintech, 66739-1-1g) (red), correlating with CEP164’s mature centriole localisation (A.I.II). Vectashield with DAPI (blue) stained cell nuclei. No primary antibody controls show no staining for CEP164 (B). (TIF) [file pone.0221914.s012.tif]
