# Supplementary material for: Embryonic and foetal expression patterns of the ciliopathy gene CEP164
Source: PLoS One. 2020 Jan 28;15(1):e0221914. doi: 10.1371/journal.pone.0221914 (PMC6986751; doi:10.1371/journal.pone.0221914)
Supplement: Attachment — Submitted filename: Devlin et al_PLoS ONE Review_2019.docx [file pone.0221914.s013.docx]

Devlin et al report embryonic and fetal expression patterns of the ciliary gene CEP164 in human and mouse specimens. CEP164 is an important distal appendage protein that localizes to the distal area of centrioles/basal bodies and its function is essential for ciliogenesis. Mutations in this gene cause NPHP-RC.

The authors performed immunohistochemistry for human embryonic sections and X-gal staining for mouse embryonic sections to conduct a thorough analysis of CEP164 expression. Similar expression patterns were observed between human and mouse tissues. CEP164 is widely expressed yet show some defined expression in multiple organs.

The study is thoroughly done and detailed expression results are provided. However, errors and mislabeling in figures make is extremely hard to read through. Although it provides new expression sites of CEP164 in embryos, it is descriptive in nature and would be suitable for a more specialized journal. Some specific points are listed below.

1) Human embryonic expression studies rely on only one antibody (Sigma). I know this has been used in several studies mostly for cultured cells, it does not necessarily mean that it is specific on tissues. I recommend to use another antibody for validation. Related to this, it would be helpful to show CEP164 localization at the base of cilia for antibody specificity as high mag images of trachea or airway for example.

2) Fig1: several rectangles for high magnification are missing. For example, A.VII, B.VII, C.VII, D.II, and E.IV. In some cases, rectangles are not accurately placed for high mag images. For example, A.V and AVI. Please also check Fig4 A.VI.

3) Fig1: it is described that CEP164 expression is seen in the cells of the uteric bud at both the apical and basal membranes as well as at the basolateral membrane of the collecting duct tubule. It is novel and interesting. However, these are very difficult to see. High mag images would be helpful. There are other places where high mag images might be beneficial such as CEP164 expression in sperm tail in Fig4.

4) In some cases where CEP164 expression is weak, for example, in NFL (Fig2A), it is hard to distinguish weak expression vs background. Perhaps, side by side comparison with negative controls may help clarify this.

Here are some errors or suggestions in the text. There are many more errors especially figure numbers so please carefully check:

P4 line 89: “basal body” to “centriole”

line 95: “Proteomics analysis” to “Protein domain analysis”

line 96: “serine/glutamine” to “serine-glutamine/threonine-glutamine”

P7 line 160: “1:1” to “1:3”

P12 line 274: remove “is”

line 275: “C.II” to “C.III”

line 283: “F.IV” to “E.IV”

line 284: “D.V” to “D.V, D.Vl” (CEP164 seems to be expressed in D.Vl)

line 284: “F.IV” to “E.II”

P14 lines 309 and 312: “P29.5” to “P30.5”
