# Supplementary material for: Embryonic and foetal expression patterns of the ciliopathy gene CEP164
Source: PLoS One. 2020 Jan 28;15(1):e0221914. doi: 10.1371/journal.pone.0221914 (PMC6986751; doi:10.1371/journal.pone.0221914)
Supplement: Attachment — Submitted filename: CEP164 rebuttalSR_JAS.docx [file pone.0221914.s014.docx]

PONE-D-19-22989

Embryonic and foetal expression patterns of the ciliopathy gene CEP164

PLOS ONE

Dear Dr Lewin,

Thank you for your comments regarding our manuscript “Embryonic and foetal expression patterns of the ciliopathy gene CEP164”. We have made amendments to the manuscript, to address the points generated in the review. Please see the revised copy of the manuscript with tracked changes, and the revised manuscript.

1) In response to “Please validate your findings by demonstrating that the primary antibody for CEP164 is specific” and “Human embryonic expression studies rely on only one antibody (Sigma). I know this has been used in several studies mostly for cultured cells, it does not necessarily mean that it is specific on tissues. I recommend to use another antibody for validation. Related to this, it would be helpful to show CEP164 localization at the base of cilia for antibody specificity as high mag images of trachea or airway for example”.

We have added a supplementary figure (S8), demonstrating that the rabbit anti-CEP164 is defined to the base of the primary cilium of human urine derived renal epithelial cells (hURECs), which are phenotypically relevant to this study and the *CEP164*-NPHP patients. Additionally we would like to emphasise that the alternative non-immunohistochemical *LacZ* reporter assay used in the murine *Cep164* expression studies further validates the human embryonic and foetal CEP164 antibody staining, due to correlating expression patterns. We therefore feel that CEP164 expression in the human embryo and foetal tissues is validated.

2) Reviewer 1s comment “Please could you quantify expression or adjust text to say that ‘expression seems to be lost with maturity” was thoroughly considered.

It was decided that quantification of CEP164 staining to compare against different time-points and tissue sub-regions could not be completed accurately enough due to the semi-quantitative nature of DAB immunohistochemistry and *LacZ* assays. Hence, the manuscript was altered so that quantification statements were not made.

3) In response to reviewer 2s comments “In some cases where CEP164 expression is weak, for example, in NFL (Fig2A), it is hard to distinguish weak expression vs background. Perhaps, side by side comparison with negative controls may help clarify this”.

Representative controls have been added to Fig 1, Fig 2 and Fig 3, to offer side-by-side comparisons. Supplementary figures S4 and S5, provide all of the controls for further analysis. Additionally, human retinal no-primary controls have been completed, to examine for endogenous peroxidase activity, and have been added to Fig 2 and S4.

4) As highlighted by reviewer 1 “Fig S1, D: not sure how this panel shows absence of CEP164 in nematoda taxa? Confusing diagram. Is there a way of labelling directly on the graph maybe?”

We acknowledge that the S1 Fig D diagram was confusing. It has been decided that it does not add sufficient information to the manuscript, and has therefore been removed.

5) All grammatical, spelling and formatting errors highlighted by the reviewers, and found within the manuscript have been resolved to the best of our knowledge. All of the Figure insets have been adjusted accordingly so that they are in the correct orientation, figures are correctly labelled and the figure panels match up with the text.
6) Reviewer 1 stated that “Black box for panel E.IV is missing, could you add it please?”, in regards to Figure 1. Additionally, reviewer 2 stated that “Fig1: several rectangles for high magnification are missing. For example…….B.VII, C.VII….and E.IV.”

There has been a misunderstanding here. The rectangles are not required in these locations due the images not being a high magnification, they are from separate medullary region of the developing kidney. This has now been clearly labelled in the figure, and figure legends.

7) Reviewer 1 stated that “Line 327: text mentions Inner Segment and Outer Segment of photoreceptor layer. Fig 2A.III does not mention these, please amend.”

Fig 2A.III has now been labelled appropriately.

8) To address the following point from reviewer 2 “Fig1: it is described that CEP164 expression is seen in the cells of the uteric bud at both the apical and basal membranes as well as at the basolateral membrane of the collecting duct tubule. It is novel and interesting. However, these are very difficult to see. High mag images would be helpful. There are other places where high mag images might be beneficial such as CEP164 expression in sperm tail in Fig4.”

Supplementary figures S3 and S7 have been generated which show digital magnification images of CEP164 expression at the nephron tubule membranes and seminiferous tubules containing sperm, respectively.

9) Reviewer 3 stated that “P7 line 160: “1:1” to “1:3””.

I feel there has been misunderstanding here. The methods are describing the mating of a heterozygous CEP164^tm1a^ mouse with a wild type mouse, therefore the ratio of HET:WT will indeed by 1:1, as we are trying to maintain the HET line and not generate HOM mice. Please see the punnet square below.

Additionally, the discussion has been modified to be more clear and concise; amendments can be seen in the tracked changes file.

The manuscript, and associated files, have been edited to meet PLOS ONE’s style requirements. All data is provided in the manuscript, and the data availability statement has been updated accordingly.

Thank you for the helpful reviews.
