# Supplementary material for: Embryonic and foetal expression patterns of the ciliopathy gene CEP164
Source: PLoS One. 2020 Jan 28;15(1):e0221914. doi: 10.1371/journal.pone.0221914 (PMC6986751; doi:10.1371/journal.pone.0221914)
Supplement: Attachment — Submitted filename: Response to Reviewers.docx [file pone.0221914.s015.docx]

**PONE-D-19-22989R1**

**Embryonic and foetal expression patterns of the ciliopathy gene CEP164**

**PLOS ONE**

Dear Dr Lewin,

Thank you for the review comments regarding our manuscript “**Embryonic and foetal expression patterns of the ciliopathy gene CEP164**”. We are pleased that you feel that the manuscript has merit, and is technically sound, with reviewer 1 recommending the manuscript for publication. We appreciate all the suggestions from the second reviewer and have made amendments to our manuscript accordingly. Please see the revised manuscript with tracked changes, and the final revised manuscript.

1) In response to;

“I have a few more edits to suggest:

line 100: remove “.”

line 126: musculus

line 171: addition

line 231: glass “

have been changed accordingly.

In response to “line 250: please add a reference here”. No reference was needed, this was referring to the DAB peroxidase staining method as mentioned in the paragraph above. The sentence on line 250 has therefore been changed into order to clarify this.

In response to “line 265: was”. This has not been changed as we believe “were” makes more grammatical sense.

2) In response to “Fig 1B V and VII are not properly cropped so please check”. Fig 1B has been corrected accordingly.
3) In response to “Letters in some figures are very hard to see for example Fig 3A VI and B VI”. The font for all of the figures has been changed to make it easier to read, with white font colour used in places to make letters easier to read.
4) In response to “The following paper can be cited: Airk et al., Roscovitine blocks collecting duct cyst growth in Cep164_deficient kidneys, Kidney Int, 2019”, this has been cited, on line 614 of the tracked changes manuscript.
